# Relationship between Brain-Derived Neurotrophic Factor and Cognitive Decline in Patients with Mild Cognitive Impairment and Dementia

**DOI:** 10.3390/biom13030570

**Published:** 2023-03-21

**Authors:** Matea Nikolac Perkovic, Fran Borovecki, Igor Filipcic, Barbara Vuic, Tina Milos, Gordana Nedic Erjavec, Marcela Konjevod, Lucija Tudor, Ninoslav Mimica, Suzana Uzun, Oliver Kozumplik, Dubravka Svob Strac, Nela Pivac

**Affiliations:** 1 Laboratory for Molecular Neuropsychiatry, Division of Molecular Medicine, Ruder Boskovic Institute, 10000 Zagreb, Croatia; 2 Department of Neurology, University Hospital Centre Zagreb, 10000 Zagreb, Croatia; 3 School of Medicine, University of Zagreb, 10000 Zagreb, Croatia; 4 Psychiatric Hospital “Sveti Ivan”, 10090 Zagreb, Croatia; 5 Department for Biological Psychiatry and Psychogeriatrics, University Psychiatric Hospital Vrapče, 10090 Zagreb, Croatia; 6 University of Applied Sciences Hrvatsko Zagorje Krapina, 49000 Krapina, Croatia

**Keywords:** Alzheimer’s disease (AD), brain-derived neurotrophic factor (BDNF), Clock Drawing test (CDT), mild cognitive impairment (MCI), Mini-Mental State Examination (MMSE)

## Abstract

In the last decade, increasing evidence has emerged linking alterations in the brain-derived neurotrophic factor (BDNF) expression with the development of Alzheimer’s disease (AD). Because of the important role of BDNF in cognition and its association with AD pathogenesis, the aim of this study was to evaluate the potential difference in plasma BDNF concentrations between subjects with mild cognitive impairment (MCI; N = 209) and AD patients (N = 295) and to determine the possible association between BDNF plasma levels and the degree of cognitive decline in these individuals. The results showed a significantly higher (*p* < 0.001) concentration of plasma BDNF in subjects with AD (1.16; 0.13–21.34) compared with individuals with MCI (0.68; 0.02–19.14). The results of the present study additionally indicated a negative correlation between cognitive functions and BDNF plasma concentrations, suggesting higher BDNF levels in subjects with more pronounced cognitive decline. The correlation analysis revealed a significant negative correlation between BDNF plasma levels and both Mini-Mental State Examination (*p* < 0.001) and Clock Drawing test (*p* < 0.001) scores. In conclusion, the results of our study point towards elevated plasma BDNF levels in AD patients compared with MCI subjects, which may be due to the body’s attempt to counteract the early and middle stages of neurodegeneration.

## 1. Introduction

Alzheimer’s disease (AD) is the most common type of dementia affecting over 20 million people worldwide [1]. This progressive neurodegenerative disease is associated with cognitive decline and impairment in memory, leading to total dependence on basic daily functions and early death. The pathological changes characteristic of AD include extracellular accumulation of amyloid β (Aβ) oligomers and the presence of intracellular neurofibrillary tangles composed of hyperphosphorylated tau proteins [2]. In addition to Aβ and tau pathology, AD patients exhibit neuronal loss in the cortical and subcortical regions, synaptic abnormalities, cognitive decline and memory loss as the disease progresses [3]. The exact cause of AD still remains unknown, but there are certain risk factors that contribute to the development of the disease. Potential risk factors for AD include aging, genetic predisposition, sex, cardiovascular factors, lifestyle, stress, head trauma, inflammatory processes, diabetes, depression, environmental factors and the presence of mild cognitive impairment (MCI) [4,5,6,7,8]. MCI is considered a transitional state between normal aging and the earliest clinical features of dementia due to its association with the increased risk of AD [9,10].

In the last decade, increasing evidence has emerged linking alterations in neurotrophic expression, in particular in the brain-derived neurotrophic factor (BDNF), with the development of AD [11,12]. BDNF belongs to the family of neurotrophic proteins, and it is important for the normal development of the central and peripheral nervous systems and plays a prominent role in the development, survival and function of neurons [13]. It modulates neurotransmission, promotes synaptic growth and regulates synaptic plasticity [14]. BDNF is expressed throughout the brain, especially in the cerebral cortex and hippocampus, regions that are essential for memory, learning and cognitive function [15]. Reduced expression of BDNF has been associated with Aβ accumulation, tau phosphorylation, neuronal apoptosis and neuroinflammation [16], which further supports the potential of BDNF as a diagnostic biomarker and therapeutic target for AD.

Alteration in BDNF signaling pathways and changes in its concentration affect memory and cognition performance in various age-related cognitive disorders, such as AD and MCI [17]. It has been suggested that BDNF levels vary with disease severity, with higher levels associated with MCI and early stages of AD and lower levels reported in patients with severe AD symptoms [18,19,20,21]. The studies investigating the association between peripheral BDNF levels and AD diagnosis reported conflicting results. While some of the studies reported decreased peripheral levels of BDNF in AD and MCI patients compared with healthy controls [15,22], other studies reported increased levels of BDNF in AD [19,23]. Either way, it is evident that BDNF is a critical factor in memory and learning processes and an important player in the processes that lead to the development of neurodegenerative diseases, such as AD.

In order to further elucidate the role of BDNF in MCI and AD, the aim of this study was to compare BDNF plasma levels between these two subject groups. This study also aimed to investigate the association between cognitive decline and plasma BDNF concentrations in patients diagnosed with AD and MCI and to assess if plasma BDNF concentrations might be used as a biomarker of cognitive decline. We hypothesized that cognitive performance and plasma BDNF concentrations would be altered in AD subjects compared with individuals diagnosed with MCI. The results of this study could lead to a better understanding of the role of BDNF in AD and strengthen the concept of BDNF as a potential diagnostic and prognostic AD biomarker in clinical practice.

## 2. Materials and Methods

### 2.1. Participants

A total of 504 participants were included in the study, 295 with AD diagnosis (40.9% male) and 209 diagnosed with MCI (40.7% male). The subjects were recruited at the University Hospital Center Zagreb (Zagreb, Croatia) and the University Psychiatric Hospital Vrapče (Zagreb, Croatia).

The diagnosis of AD was based on DSM-5 [24] criteria and the criteria of the National Institute of Neurological and Communication Disorders and Stroke, which is part of the American National Institute of Health (NINCDS-ADRDA; National Institute of Neurological and Communicative Disorders and Stroke and Alzheimer’s Disease and Related Disorders Association) [25]. MCI was diagnosed using the criteria defined by Petersen et al. [26] and Albert and colleagues [9]. The average age of onset for AD patients was 66.8 ± 9.9 years, and for subjects diagnosed with MCI, 63.0 ± 10.6 years. The duration of the disease was, on average, 2.5 ± 1.8 years for AD subjects and 2.1 ± 1.5 years for individuals with MCI. The cognitive abilities of all participants were evaluated using the Mini-Mental State Examination (MMSE) [27,28] and the Clock Drawing test (CDT) [29,30].

All participants included in the study were in- and out-patients who have undergone neurological examination, thyroid function examination and serologic tests for Lyme disease and syphilis. The levels of vitamin B12 and B9 were also determined for the participants included in the study. The exclusion criteria for both groups of subjects were alcohol dependence or heavy alcohol use in order to exclude alcohol-induced cognitive deficits. The subjects were not related to each other and had not been previously prescribed any antidementia medication. Subjects diagnosed with vascular or mixed dementia, tumors or inflammatory diseases of the central nervous system, brain trauma, systemic metabolic diseases and other psychiatric or neurological diseases (e.g., Huntington’s disease, Frontotemporal dementia) were excluded from the study.

This study was carried out in line with the Helsinki Declaration [31], and it was approved by the Ethical Committee of the Clinical Hospital Center Zagreb, Croatia (case no. 02/21 AG, class 8.1-18/82-2 from 24 April 2018) and by the Ethics Committee of the University Psychiatric Hospital Vrapče, Zagreb, Croatia (approval code 23-605/3-18; 23 March 2018). Study procedures were explained in detail to the patients and their caregivers. All participants have signed informed consent prior to participating in the study.

### 2.2. Blood Sample Collection

Blood samples (8.5 mL) were collected after an overnight fast, in the morning hours between 7.00 AM and 9.00 AM during the routine laboratory examination, in yellow-top BD Vacutainer™ tubes (Becton, Dickinson and Company, Franklin Lakes, NJ, USA) with 1.5 mL of acid citrate dextrose anticoagulant. Plasma was separated from the whole blood samples by centrifugation (3 min at 1100× *g*, followed by 15 min at 5030× *g*). Plasma samples were stored at −20 °C until further analysis.

### 2.3. Measurement of Plasma BDNF Concentration

The concentration of BDNF in plasma was determined with a commercial enzyme-linked immunosorbent assay (ELISA) according to the manufacturer’s instructions (Quantikine ELISA, R&D Systems, Minneapolis, MN, USA). All samples were measured in duplicates and diluted 1:2 with a dilutant provided by the manufacturer. Plasma samples and standards of appropriate concentration were added into 96-well plates pre-coated with the monoclonal antibody specific for human BDNF. The plates were incubated at room temperature for 2 h. A monoclonal antibody, which is specific for human BDNF and conjugated to horseradish peroxidase, was added to each well. The plates were again incubated at room temperature for 1 h. Following the incubation, plates were washed 3 times with washing buffer in order to remove any unbound antibody–enzyme reagent. After adding the substrate solution, the plates were incubated at room temperature for 30 min, protected from light. The reaction was stopped by adding 2 N sulfuric acid. The absorbance of each sample, standards and blanks was measured using a microplate reader set to 450 nm with wavelength correction set to 570 nm. The intra- and inter-assay coefficients of variations were less than 10%. The concentrations of samples in each plate were calculated based on a standard curve.

### 2.4. Statistical Analysis

The results were evaluated with Sigma Stat 3.5 (Jandel Scientific Corp., San Jose, CA, USA). The Kolmogorov–Smirnov test was used to assess the normality of data distribution for all the variables included in the study (BDNF plasma concentration, demographic and clinical variables). Due to the deviation from a normal distribution, non-parametric tests were applied for all the statistical analyses, and the results were expressed as median and range (minimum–maximum). The Mann–Whitney U test was used to compare two groups of subjects, and the Kruskal–Wallis ANOVA, by ranks with Dunn’s multiple comparisons test for post hoc comparisons, was used to compare subjects after dividing them into categories according to MMSE scores. Correlations between BDNF plasma concentrations and demographic and clinical parameters were performed using Spearman’s correlation coefficient. All tests were two-tailed, and α was set at 0.05.

G*Power version 3.1.9.4. software [32] was used to calculate the needed sample size and statistical power. With expected effect size = 0.30, statistical power = 0.80 and α error probability = 0.05, the required sample size was n = 384 for the Mann–Whitney U test, n = 128 for Kruskal–Wallis ANOVA and n = 370 for correlation analyses. Because the study included 504 participants, it had the needed sample size to detect differences between subject groups.

## 3. Results

### 3.1. Participants

The study included 504 subjects (40.8% male) who were divided into subjects with AD (n = 295) and subjects with MCI (n = 209). These two groups have a similar proportion of male and female participants (χ^2^ = 0.00; df = 1; *p* = 0.958). Other demographic and clinical characteristics of the participants are shown in Table 1. Due to the deviation of all examined demographic and clinical parameters from a normal distribution, non-parametric statistical tests were used to compare the data between subject groups (Table 1).

Two groups of subjects significantly differ (Table 1) in age (*p* < 0.001) and cognitive abilities assessed with MMSE (*p* < 0.001) and CDT (*p* < 0.001). Namely, patients with AD are significantly older and have significantly lower MMSE and CDT scores than subjects with MCI. Subjects have similar BMIs (*p* = 0.354), waist circumferences (*p* = 0.986), total cholesterol levels (*p* = 0.566), HDL- (*p* = 0.888) and LDL-cholesterol (*p* = 0.278) concentrations, triglycerides (*p* = 0.569) and blood glucose levels (*p* = 0.153).

### 3.2. Plasma BDNF Concentration in MCI Subjects and AD Patients

Plasma BDNF concentrations did not fit a normal distribution; therefore, non-parametric statistical methods were used for analysis that included data on BDNF plasma concentrations.

Due to the potential influence of demographic and clinical characteristics on plasma BDNF concentrations, the correlation between BDNF plasma concentrations and specific demographic and clinical parameters was assessed in both groups of subjects using Spearman’s correlation coefficient (Table 2). The results show no significant correlations between plasma BDNF levels and demographic and clinical parameters in subjects with MCI and patients diagnosed with AD (Table 2).

The comparison of BDNF plasma concentration between male and female subjects reveals no significant difference in BDNF plasma levels between male and female subjects with MCI (U = 5598.0; *p* = 0.445) and between male and female patients with AD (U = 917.0; *p* = 0.218).

Due to these results, there was no need to include age, sex, BMI, waist circumference, total cholesterol, HDL-cholesterol, LDL-cholesterol, triglycerides and fasting glucose values in further analyses.

**Figure 1:**
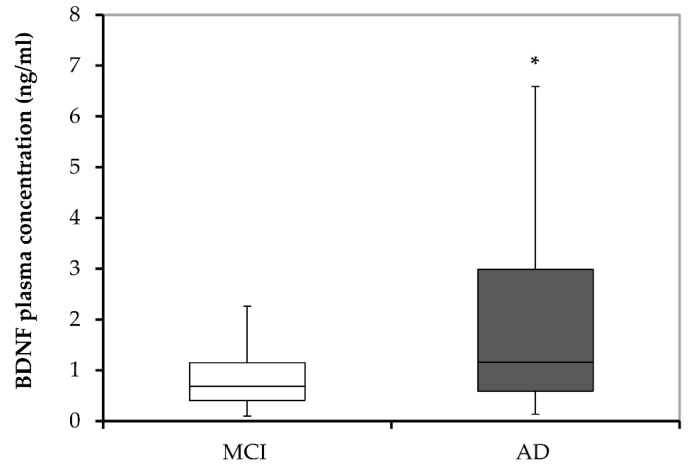
BDNF plasma concentrations in MCI subjects and patients diagnosed with AD. AD, Alzheimer’s disease; BDNF, brain-derived neurotrophic factor; and MCI, mild cognitive impairment. * *p* < 0.001 vs. MCI.

The level of plasma BDNF was compared between subjects with MCI and patients with AD to explore the possible association of BDNF plasma concentration with the diagnosis of AD (Figure 1). The results reveal significantly higher (U = 40,449.0; *p* < 0.001) concentrations of plasma BDNF in subjects with AD (1.16; 0.13–21.34) compared with individuals with MCI (0.68; 0.02–19.14).

### 3.3. Plasma BDNF Concentrations and Cognitive Decline

In order to assess the association of plasma BDNF concentrations and cognitive decline in subjects with MCI and patients diagnosed with AD, Spearman’s correlation coefficient was used. The correlation analysis reveals a significant (although weak) negative correlation between BDNF plasma levels and both MMSE (r_s_ = −0.219; *p* < 0.001) and CDT (r_s_ = −0.204; *p* < 0.001) scores in our combined sample that consisted of 504 subjects diagnosed with MCI or AD.

**Figure 2:**
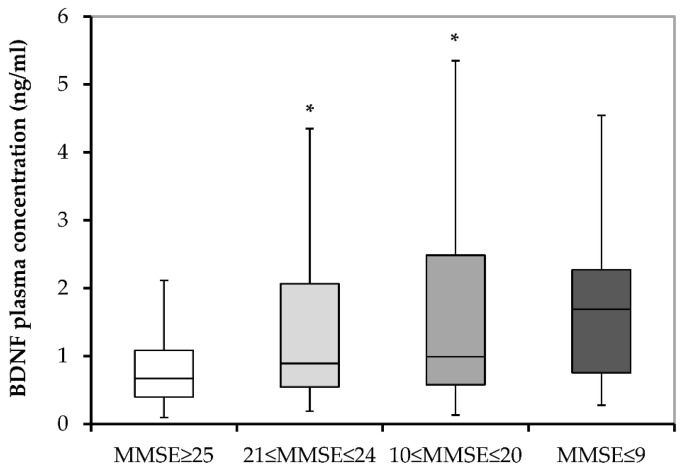
BDNF plasma concentrations in all subjects subdivided into those with normal cognition (MMSE ≥ 25), those with mild dementia (21 ≤ MMSE ≤ 24), those with moderate dementia (10 ≤ MMSE ≤ 20) and those with severe dementia (MMSE ≤ 9). BDNF, brain-derived neurotrophic factor; and MMSE, Mini-Mental State Examination. * *p* ≤ 0.050 vs. MMSE ≥ 25.

In order to confirm this negative association, we additionally divided all subjects (both patients with AD and subjects with MCI) into categories according to the MMSE and CDT scores. According to the MMSE scores, subjects were subdivided into those with normal cognition (with 25–30 MMSE scores; N = 185), those with mild dementia (with 21–24 MMSE scores; N = 133), those with moderate dementia (with 10–20 MMSE scores; N = 174) and those with severe dementia (with 0–9 MMSE scores; N = 12). Significantly higher (H = 19,217.0; *p* < 0.001) plasma BDNF concentrations are detected in subjects with moderate (*p* < 0.001; Dunn’s post hoc test) and mild (*p* = 0.023; Dunn’s post hoc test) dementia compared with subjects with normal cognition (Figure 2). In the case of subjects with severe dementia, a similar trend is observed (i.e., higher BDNF levels vs. subjects with MMSE ≥ 25), but the increase in BDNF plasma concentrations is not statistically significant (*p* = 0.056; Dunn’s post hoc test).

**Figure 3:**
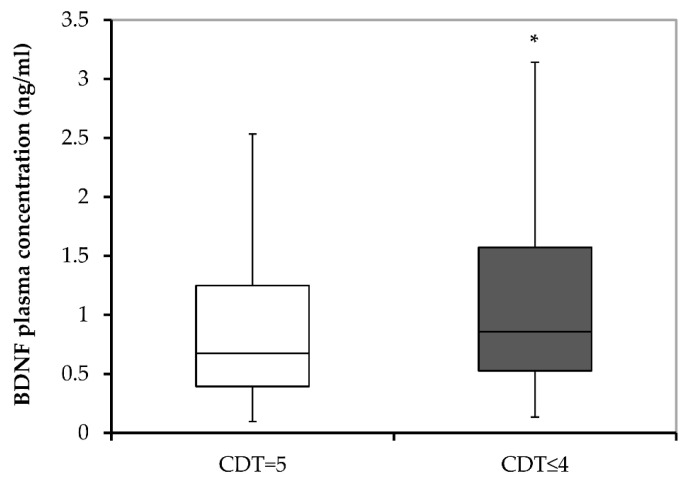
BDNF plasma concentrations in subjects subdivided into those with normal cognition (CDT = 5) and those with cognitive disturbances (CDT ≤ 4). BDNF, brain-derived neurotrophic factor; and CDT, Clock Drawing test. * *p* < 0.001 vs. CDT = 5.

According to the CDT scores, combined groups of all subjects were subdivided into two groups, those with normal cognition (with 5 CDT scores; N = 250) and those with cognitive disturbances (with 0–4 CDT scores; N = 254). Plasma BDNF concentration significantly differed (U = 14,534.0; *p* = 0.010) due to the significantly higher levels of plasma BDNF levels in subjects with cognitive disturbances compared with subjects with normal cognition (Figure 3).

## 4. Discussion

The main findings of this study are (1) increased plasma BDNF concentrations in AD patients compared with MCI subjects; (2) a negative correlation between cognitive decline and plasma BDNF concentrations; and (3) higher BDNF concentrations in subjects with mild and moderate cognitive decline compared with subjects with normal cognition.

The increasing number of people with advanced AD represents a significant emotional and financial burden on the global population, making early detection of preclinical AD critical to the success of prevention and treatment strategies. Biomarkers, which are measurable characteristics and objective indicators of normal and pathogenic processes, as well as markers of treatment effectiveness [33], play a vital role in this attempt. The search for peripheral biomarkers for AD is based on the idea that the changes visible in the CNS can also be detected in the peripheral tissues [34]. Because of the important role of BDNF in cognition and its association with AD pathogenesis, the aim of this study was to evaluate the potential difference in plasma BDNF concentrations between subjects with MCI and AD patients and to determine the possible association between BDNF plasma levels and the degree of cognitive decline in these individuals.

Studies have shown that BDNF can cross the blood–brain barrier [35,36,37], and a positive correlation between peripheral BDNF levels and cortical regions in animals has been confirmed [38]. Platelets are one of the major sources of BDNF in the periphery, with the ability to bind, store and release BDNF for repair [39], suggesting that an increase in BDNF levels may occur due to damage or injury. Serum BDNF levels are influenced by the amount of BDNF stored in platelets, whereas plasma BDNF levels reflect the current state of the organism and the amount of bioactive protein available [39]. Therefore, BDNF concentration in plasma is a more objective indicator of an organism’s condition than its concentration in serum. Some authors even suggested that the increased serum BDNF concentration may be related to the inflammatory process visible in patients with AD [19]. In this study, BDNF concentrations in plasma were used instead of serum to avoid the possible influence of BDNF released by platelets [39] and immune cells [40]. Studies have suggested that plasma BDNF levels may be affected by age [41,42]. In cases where differences in peripheral BDNF levels have been observed between males and females, these changes are thought to be related to estrogen secretion [41,43,44]. Estrogens may be involved in the regulation of BDNF expression, and there is also an overlap in the action of these two molecules [45,46]. BDNF concentration is thought to fluctuate less later in life, and the influence of estrogen on BDNF concentration is lost in older women. In our study, we examined the correlation between plasma BDNF concentrations and certain demographic and clinical characteristics to rule out their possible influence on plasma BDNF concentrations. We found no correlation with any of the examined variables, including age. Additionally, we did not detect any difference in plasma BDNF concentrations between male and female subjects.

The determination of plasma BDNF concentrations as a blood-based biomarker has attracted considerable attention over the past decade. Research focusing on the relationship between peripheral BDNF concentration and AD has yielded mixed results, with some studies reporting decreased levels in AD patients [4,5,6,7,8,9,10,11,12,13,14,15,16,17,18,19,20,20,21,22,23,24,25,26,27,28,29,30,31,32,33,34,35,36,37,38,39,40,41,42,43,44,45,46,47,48,49,50,51,52,53] and others finding no difference in BDNF concentrations [54] or even increased concentrations [19,50,51,52,53,54,55]. Our results suggest significantly increased plasma BDNF concentration in AD subjects compared with individuals diagnosed with MCI. We have observed a similar trend in our previous study, which focused on the difference in plasma BDNF concentrations between veterans with combat-related post-traumatic stress disorder (PTSD) and healthy controls [56]. In that study, we found an interesting trend toward higher concentrations of plasma BDNF in AD patients compared with the MCI group [56], which prompted us to study in more detail the alterations of plasma BDNF levels in individuals with AD compared to subjects with MCI and to further investigate the association of BDNF concentrations with the level of cognitive impairment in these subjects. In order to confirm that, in this study, we have found the same trend that we saw in the previous study [56], and to eliminate potential differences between the groups of subjects with AD or MCI included in these two studies, we made additional comparisons between the mentioned subjects. Comparing plasma BDNF concentrations between subjects diagnosed with MCI from both studies revealed no significant difference (U = 5327.00; *p* = 0.364) in plasma BDNF levels between the MCI group from our previous study (0.68; 0.02–19.14) and a larger group of MCI subjects (0.80; 0.14–13.04) in the present study. Comparison between AD subjects from our previous study (1.16; 0.13–21.34) and a much larger group of AD patients from the current study (1.07; 0.17–17.59) also revealed no significant difference in BDNF plasma concentration (U = 10,502.00; *p* = 0.396). However, the trend of reduced BDNF plasma concentration in subjects with AD diagnosis, compared to individuals with MCI, was confirmed by comparing the smaller group of MCI subjects from the previous study by Domitrovic Spudic et al. [56] and the AD group from the present study (U = 5468.00; *p* = 0.020), and by comparing AD subjects from our former study with MCI group in this study (U = 10,155.00; *p* < 0.001). These results further strengthen our hypothesis and confirm a similar trend in both studies. The comparison between the two cohorts (results from the previous study by Domitrovic Spudic et al. [56] and the current study) is presented in Supplementary Materials (Figure S1). As expected, all four groups of subjects significantly differed (H = 71.84; df = 4; *p* ≤ 0.001) in plasma BDNF concentration from BDNF values in healthy control subjects (1.74; 0.15–20.33) that were included in our former study (Supplementary Materials, Figure S1). The comparison showed significantly higher levels of plasma BDNF in healthy subjects compared with all other participants. However, we have to point out that the healthy control subjects in our former study [56] were age-matched to PTSD patients and were, therefore, much younger than both MCI and AD groups. As mentioned, the results confirmed significantly higher concentrations of BDNF in the plasma samples of subjects diagnosed with AD (both cohorts) compared with subjects diagnosed with MCI (Supplementary Materials, Figure S1). Lack of significant difference in plasma BDNF levels between AD patients and MCI subjects in the first cohort is probably due to a smaller sample size of subject groups that were included in our previous study [56]. In contrast, the present study included much larger groups of subjects with MCI and AD, without healthy controls, and age was not significantly correlated with plasma BDNF concentrations.

In disagreement with our previous data [56], the results of the present study revealed a significant negative correlation between cognitive functions and BDNF plasma concentration, suggesting higher BDNF levels in subjects with more pronounced cognitive decline. Discrepancies between present and previous [56] results might be due to the inclusion of diagnostic categories (PTSD, healthy groups in our previous study [56]) and the lack of inclusion of the older healthy group with normal cognition in the present study. The present study included only cognitively more- or less-impaired subjects. Other studies on the role of BDNF in the development of cognitive symptoms in dementia have yielded conflicting results [56,57,58,59,60]. Previous studies observed a positive association between BDNF blood levels and cognitive performance in healthy elder subjects and AD patients [43,61]. Siuda and colleagues demonstrated a significant correlation between BDNF serum levels and cognitive impairment, where lower levels were observed in AD subjects [53]. In contrast, other studies reported no association between BDNF serum levels and MMSE scores in AD patients [19,22,47,62]. These discrepancies could be explained by the differences in diagnostic criteria, patient recruitment and stages of diseases [19]. Meta-analysis has shown that peripheral BDNF levels gradually decrease with increasing cognitive impairment [63] and are only detectable in late-stage disease [15]. It has been reported that a decrease in cerebrospinal fluid BDNF levels may be associated with cognitive decline in healthy individuals [60]. Regarding serum BDNF concentrations, some results confirm a link between BDNF and better cognitive performance in healthy individuals [63], and others do not corroborate this association [57]. A study by Shimada et al. [64] suggested an association between lower serum BDNF concentration, diagnosis of MCI and poor cognitive ability in the elderly. As for the plasma BDNF, there are data that do not support its role in the development of cognitive symptoms [58], and other findings report an association between cognitive function and BDNF in subjects after adjusting for the influence of multiple cofactors [43]. Our results showed that plasma BDNF levels were increased in AD patients compared with subjects with MCI. A negative correlation between plasma BDNF concentrations and psychometric test scores (MMSE and CDT) was detected when the relationship between plasma BDNF concentrations and cognitive decline was examined in all subjects (i.e., a combined group with AD and MCI). All subjects were categorized according to MMSE (normal cognition, mild, moderate, and severe dementia) and CDT (normal cognition and cognitive disturbances) scores to confirm this negative correlation. When looking at MMSE scores, BDNF plasma concentrations were significantly higher in subjects with mild and moderate dementia than in subjects with normal cognition. A similar trend was observed in subjects with severe dementia, in whom BDNF plasma concentrations were increased compared with subjects with normal cognition, but this result was not statistically significant. Similarly, the results, which rely on CDT scores for the assessment of the level of cognitive impairment, also demonstrated a significant difference in BDNF plasma concentrations between subjects with normal cognition and individuals with cognitive disturbances, with plasma BDNF concentration being higher in subjects with cognitive impairment. In contrast to most studies that used only the MMSE test to assess cognitive impairment, our study has combined the MMSE and CDT. The CDT is commonly used to measure cognitive impairment in patients with dementia. Given the limitations of each psychometric test used to assess dementia, a combination of different psychometric tests was recommended in order to improve the detection of individuals with MCI and the early stage [65,66]. Consistent with our findings, a similar pattern of BDNF alterations has been observed in the early stages of AD, characterized by a decrease in BDNF levels, followed by an increase [20,67]. Other studies have shown a gradual increase of BDNF levels associated with severity of cognitive impairment [68,69]. This trend was also supported by the results of Laske et al. [18], Angelucci et al. [19] and Faria and colleagues [55]. The observed results could be the consequence of a compensatory repair mechanisms underlying the upregulation of BDNF expression, as an attempt to compensate for the negative effects of the disease on neuronal degeneration and increased accumulation of Aβ in the CNS [18]. Kim and colleagues reported that peripheral BDNF levels in AD patients initially increase in the early stages of the disease compared with healthy, age- and sex-matched controls, and then decrease in patients with moderate or severe AD [21]. The initial increase in peripheral BDNF levels in the early stages of AD may be due to compensatory repair mechanisms, and, as the disease progresses and becomes more severe (indicated by a low MMSE and CDT scores), these compensatory mechanisms may begin to fail, leading to a decrease in peripheral BDNF levels. In addition to its function as a potential repair mechanism for the early and late stages of neurodegeneration, the increase in BDNF may also have a neuroprotective effect through its involvement in Aβ degradation [19]. In support of this hypothesis, some studies have shown that BDNF can promote Aβ degradation by enhancing the expression of somatostatin [70,71]. Furthermore, BDNF may deactivate glycogen synthase kinase-3 beta (GSK3β) [72], which plays a role in the abnormal phosphorylation of tau protein [73]. Another possible explanation for this accumulation could be a disruption of axonal transport or utilization of BDNF in the CNS, leading to increased levels of BDNF in the bloodstream [19]. The results of our study show that AD patients have elevated plasma BDNF concentration compared with MCI subjects, which may be due to the body’s attempt to counteract the early and middle stages of neurodegeneration. Numerous research studies have reported that peripheral BDNF concentrations can be a valuable indicator for diagnosing, predicting disease progression and monitoring the effectiveness of AD treatment. However, the results of different studies, regarding the peripheral BDNF concentration in AD patients, are highly variable [63,74,75,76,77]. In addition, clinical studies imply that BDNF can serve as both a biomarker and therapeutic target in AD, and there are propositions for using composite biomarkers, such as dual-specificity tyrosine phosphorylation-regulated kinase 1A (DYRK1A), homocysteine and BDNF, to facilitate the early diagnosis of AD [78].

The main limitations of this study are its cross-sectional design and the fact that we were not able to include a healthy control group that would be age-matched with older subjects diagnosed with AD or MCI.

The strengths of the study are the adequate sample size (n = 504) and the needed statistical power, along with the inclusion of ethnically homogenous groups. An additional advantage of the study is that BDNF plasma concentrations were determined by investigators who were blind to the diagnoses of subjects and by using R&D System-Quantikine BDNF ELISA assays, which were reported to be specific for mature human mature BDNF [78,79].

In conclusion, this study found elevated plasma BDNF concentrations in AD patients compared with MCI subjects and a negative correlation between cognitive falls and plasma BDNF levels. Higher BDNF concentration in subjects with more severe cognitive decline in the combined group of subjects with AD and MCI might suggest a possible compensatory repair mechanism causing the upregulation of BDNF expression in order to counteract the highest burden of AD pathology.

## Figures and Tables

**Table 1:** Demographic and clinical characteristics of individuals diagnosed with MCI and AD patients. All data are presented as median (range).

	Participants		Mann–Whitney U Test	
	MCI	AD	U	*p*
Age (years)	71.0 (41.0–88.0)	77.5 (49–94)	32,536.5	<0.001
BMI (kg/m^2^)	22.0 (18.4–32.4)	23.4 (18.5–31.9)	6935.5	0.354
Waist circumference (cm)	86.0 (71.0–101.0)	86.0 (72.0–99.0)	6459.0	0.986
Total cholesterol (mmol/L)	5.4 (3.2–8.8)	5.7 (3.2–8.8)	6759.0	0.566
HDL-cholesterol (mmol/L)	1.3 (0.7–3.0)	1.3 (0.7–3.0)	6397.0	0.888
LDL-cholesterol (mmol/L)	3.1 (0.8–5.6)	3.3 (0.8–5.8)	7016.0	0.278
Triglycerides	1.8 (0.7–6.7)	1.8 (0.7–6.7)	6756.0	0.569
Blood glucose (mmol/L)	5.5 (4.4–11.8)	5.6 (4.7–11.8)	7191.5	0.153
MMSE score	27.0 (21.0–30.0)	16.5 (0.0–27.0)	1535.5	<0.001
CDT score	5.0 (0.0–5.0)	1.0 (0.0–5.0)	7129.0	<0.001

AD, Alzheimer’s disease; CDT, Clock Drawing test; HDL, high-density lipoproteins; BMI, body mass index; LDL, low-density lipoproteins; MCI, mild cognitive impairment; MMSE, Mini-Mental State Examination; and N, number of participants.

**Table 2:** Correlation of plasma BDNF concentrations with demographic and clinical characteristics of subjects diagnosed with MCI or AD.

Characteristics	MCI		AD	
	r_s_	*p*	r_s_	*p*
Age (years)	−0.135	0.057	0.126	0.057
BMI (kg/m^2^)	0.011	0.891	0.055	0.618
Waist circumference (cm)	0.009	0.910	0.071	0.523
Total cholesterol (mmol/l)	−0.037	0.646	−0.151	0.170
HDL-cholesterol (mmol/l)	−0.083	0.308	−0.092	0.406
LDL-cholesterol (mmol/l)	−0.040	0.624	−0.088	0.427
Triglycerides	0.024	0.764	0.021	0.850
Blood glucose (mmol/l)	−0.024	0.765	0.204	0.063

AD, Alzheimer’s disease; HDL, high-density lipoproteins; BMI, body mass index; LDL, low-density lipoproteins; MCI, mild cognitive impairment; and r_s_, Spearman’s rank correlation coefficient.

## Data Availability

All the data reported are available upon request from the corresponding author.

## Supplementary Materials

The following supporting information can be downloaded at: https://www.mdpi.com/article/10.3390/biom13030570/s1, Figure S1: Comparison of the results (Kruskal-Wallis ANOVA by ranks), concerning BDNF plasma concentration in MCI subjects and patients diagnosed with AD, between current study (cohort 2) and previous study by Domitrovic Sudic and colleagues [56] (cohort 1).
