# Supplementary material for: Relationship between Brain-Derived Neurotrophic Factor and Cognitive Decline in Patients with Mild Cognitive Impairment and Dementia
Source: Biomolecules. 2023 Mar 21;13(3):570. doi: 10.3390/biom13030570 (PMC10046678; doi:10.3390/biom13030570)
Supplement: Supplementary file 1 [file biomolecules-13-00570-s001.zip › biomolecules-2256381-supplementary.pdf]

biomolecules

MDPI

Article

Relationship between Brain-Derived Neurotrophic Factor and Cognitive Decline in Patients with Mild Cognitive Impairment and Dementia

Matea Nikolac Perkovic [1], Fran Borovecki [2,3], Igor Filipcic [4], Barbara Vuic [1], Tina Milos [1], Gordana Nedic Erjavec [1], Marcela Konjevod [1], Lucija Tudor [1], Ninoslav Mimica [3,5], Suzana Uzun [3,5], Oliver Kozumplik [5], Dubravka Svob Strac [1] and Nela Pivac [1,6,*]

[1] Laboratory for Molecular Neuropsychiatry, Division of Molecular Medicine, Ruder Boskovic Institute, 10000 Zagreb, Croatia; mnikolac@irb.hr (M.N.P.); barbara.vuic@irb.hr (B.V.); tina.milos@irb.hr (T.M.); gnedic@irb.hr (G.N.E.); mkonjev@irb.hr (M.K.); ltudor@irb.hr (L.T.); dsvob@irb.hr (D.S.S.)
[2] Department of Neurology, University Hospital Centre Zagreb, 10000 Zagreb, Croatia; fbor@mef.hr
[3] School of Medicine, University of Zagreb, 10000 Zagreb, Croatia; ninoslav.mimica@bolnica-vrapce.hr (N.M.); suzana.uzun@bolnica-vrapce.hr (S.U.)
[4] Psychiatric Hospital "Sveti Ivan", 10090 Zagreb, Croatia; igor.filipcic@pbsvi.hr
[5] Department for Biological Psychiatry and Psychogeriatrics, University Psychiatric Hospital Vrapče, 10090 Zagreb, Croatia; oliver.kozumplik@bolnica-vrapce.hr
[6] University of Applied Sciences Hrvatsko Zagorje Krapina, 49000 Krapina, Croatia
* Correspondence: npivac@irb.hr

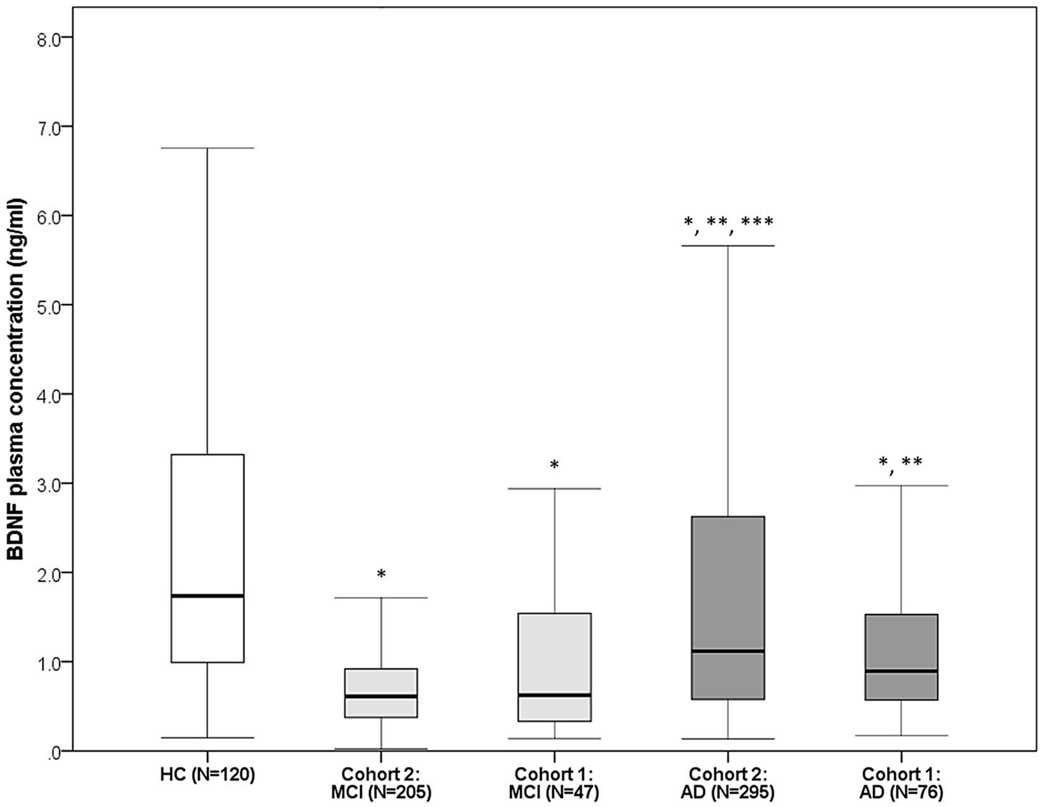

Figure S1. Comparison of the results (Kruskal-Wallis ANOVA by ranks), concerning BDNF plasma concentration in MCI subjects and patients diagnosed with AD, between current study (cohort 2) and previous study by Domitrovic Sudic and colleagues [59] (cohort 1).
AD, Alzheimer's Disease; BDNF, Brain-Derived Neurotrophic Factor; HC, Healthy Control; MCI, Mild Cognitive Impairment

* p≤0.001 vs. HC (Dunn's post-hoc test)

** p≤0.001 vs. MCI, Cohort 2 (Dunn's post-hoc test)

*** p<0.050 vs. MCI, Cohort 1 (Dunn's post-hoc test)
